# The Therapeutic Properties of Plants Used Traditionally to Treat Gastrointestinal Disorders on Groote Eylandt, Australia

**DOI:** 10.1155/2020/2438491

**Published:** 2020-11-10

**Authors:** Cécile Mazerand, Ian Edwin Cock

**Affiliations:** ^1^School of Environment and Science, Griffith University, Brisbane 4111, Australia; ^2^School of Biology, Ecole de Biologie Industrielle (EBI), Cergy, France; ^3^Environmental Futures Research Institute, Griffith University, Brisbane, Australia

## Abstract

The First Australians had well-developed healing systems. Groote Eylandt inhabitants used a variety of plant species to treat diarrhoea and other gastrointestinal illnesses. This study was undertaken to test, identify, and evaluate traditional medicines to treat these conditions against gastrointestinal bacterial, protozoal, and viral pathogens, as well as against cancer cell proliferation. Six plant species (*Buchanania obovata* Engl., *Casuarina equisetifolia* L., *Eucalyptus tetrodonta* F. Muell., *Planchonia careya* (F. Muell.) R. Knuth, *Terminalia carpentariae* C. T. White, and *Vigna vexillata* (L.) A. Rich.) were selected from a survey of a panel of elders from the Warnindhilyagwa tribe and compared with the published literature. Decoctions prepared according to traditional methods were screened for growth inhibitory activity of a panel of diarrhoea-causing bacterial pathogens by disc diffusion and liquid dilution MIC assays. Inhibitory activity against the gastrointestinal protozoal parasite *Giardia duodenalis* and antiproliferative activity against human colorectal (Caco2) and cervical (HeLa) cancer cell lines were evaluated using MTS-based colorimetric cell proliferation assays. Preliminary antiviral screening was accomplished using an MS2 bacteriophage plaque reduction assay. Toxicity was evaluated using *Artemia franciscana* nauplii mortality and HDF cell viability bioassays. All traditional medicines tested inhibited bacterial growth, often with MIC values substantially <1000 *μ*g/mL. *T. carpentariae* was particularly noteworthy, with MIC values of 230–350 *μ*g/mL against *Citrobacter freundii*, *Salmonella newport*, *Shigella sonnei*, *Staphylococcus aureus*, and *Staphylococcus epidermidis*. This species also had MICs 450–950 *μ*g/mL against all other bacterial pathogens. *B. obovata* Engl. and *E. tetrodonta* were also good inhibitors of bacterial growth, albeit with substantially higher MIC values than determined for *T. carpentariae.* The *T. carpentariae* decoction was also the best inhibitor of MS2 phage replication (IC_50_ = 427 *μ*g/mL) and Caco2 and HeLa proliferation (IC_50_ values of 885 and 85 *μ*g/mL, respectively). None of the extracts were particularly strong inhibitors of *Giardia duodenalis* growth. All decoctions were nontoxic in the *Artemia* nauplii and HDF cell viability bioassays, indicating their suitability for therapeutic use.

## 1. Introduction

The World Health Organization (WHO) has estimated that nearly nine million children under the age of five die every year as a result of diarrhoea [1]. Despite that report being more than a decade old, very little has changed in the interim, and diarrhoea remains the leading killer of children globally, accounting for approximately 9% of all deaths among children under the age of five [2]. This translates into more than 1400 young children dying each day, or about 530,000 children a year. To exacerbate this problem, many bacteria have developed resistance to conventional antibiotics, rendering them of little use against some diarrhoea-causing pathogens [3]. There is an urgent need to develop new treatment options to combat these diseases through the development of novel drugs.

Plants have long been used in traditional healing systems to treat diarrhoea. These traditional medicines may be given as single-component therapies or they may be prescribed in combination to target the multiple negative effects of diarrhoea (loose stools, cramps, loss of electrolytes, and possible fever). The activity of several of the herbal preparations used traditionally to treat diarrhoea and other gastrointestinal diseases has already been validated by rigorous scientific evaluation. This is particularly true for plant medicines used in traditional Indian healing systems (including Ayurveda, Siddha, and Unani) [4–6] and in traditional Chinese medicine (TCM) [7, 8]. In contrast, the potential of many Australian medicinal plants in alleviating the symptoms of diarrhoea and inhibiting the pathogenic causes has been relatively neglected.

Despite being one of the oldest continuous civilisations globally, far less information is available describing the ethnobotany of the First Australians than for many other ethnic groups globally. The First Australians had a good understanding of the medicinal properties of Australian plants and had used them successfully for at least 50,000 years to treat a variety of illnesses. However, little of this information has been recorded as the First Australians did not have a written language. Instead, traditional knowledge was passed between generations orally, and most ethnobotanical texts have collated data from the perspective of European settlers. As a result, substantial ethnobotanical information has been irretrievably lost. Furthermore, many of the seminal texts that summarise Aboriginal plant usage are old and difficult to access, taxonomic classifications are outdated and often dubious, and the information is incomplete. Compounding the problem, the First Australians consist of diverse groups, with individual communities having their own language, culture, customs, and belief systems, as well as their own traditional knowledge. Members of one language group were generally unable to communicate effectively with other groups. Within the language groupings, the First Australians are further divided into local groupings, which often had regional dialects, further complicating communication. Furthermore, many of the First Australian groups lived in isolation from other groups. As such, exchange of knowledge between groups was often not viable. Additionally, Torres Strait Islanders have distinct heritage from the Aborigines, with substantially different customs and traditional knowledge. Thus, plants used traditionally by one group of First Australians may not have been used for the same purposes by other groups. Despite this relative dearth of rigorous ethnobotanical studies on Australian flora, several studies have identified plant species with noteworthy antimicrobial activity. In particular, the ethnobotany of the inhabitants of Groote Eylandt has been relatively well recorded, allowing for selection of relevant species to treat microbial pathogens [9].

Bacterial pathogens are major causes of diarrhoea, and several studies have already screened Australian medicinal plants for antidiarrheal properties. Palombo and Semple investigated the growth inhibitory properties of extracts prepared from 39 Australian plant species against a panel of bacterial pathogens, including several diarrhoea-causing species [10]. That study reported that several species had noteworthy inhibitory activity and highlighted *Eremophila duttonii* F. Muell. as having the greatest activity of the species tested. However, that study screened the plant extracts at a single dose (concentration not specified), and MIC values were not reported, making a comparison with other studies impossible. Furthermore, the Palombo and Semple study tested ethanolic extracts instead of the decoctions that were used by the First Australians. Therefore, whilst that study was interesting and highlighted several plants worthy of further evaluation, it did not validate the traditional use of those species to treat diarrhoea. Another study screened extracts prepared from 109 Australian plants for the ability to inhibit the growth of *Campylobacter jejuni*, which is one of the most common causes of acute enteritis in humans, and reported potent activity for many species [11]. That study reported MIC values below 1000 *μ*g/mL for all species against two *C. jejuni* strains and similar potency against several other diarrhoea-causing bacteria. Several *Eucalyptus* spp. were amongst the most potent species tested in that study. Several recent studies have also reported good inhibitory activity for several high-antioxidant Australian plant species against multiple bacterial species, including diarrhoea-causing pathogens [12–14]. A recent study also tested *Terminalia ferdinandiana* Excell. extracts against multiresistant ESBL *Escherichia coli* and methicillin-resistant *Staphylococcus aureus* (MRSA) and reported MIC values as low as 200 *μ*g/mL [15]. Several studies have also reported good inhibitory activity for Australian plants against the gastrointestinal protozoal parasite *Giardia duodenalis* [16–18]. Furthermore, a recent study from our group reported notable antimicrobial activity for one of the species examined herein (*B. obovata*) [19, 20]. However, all those studies examined extracts prepared under laboratory conditions using organic solvents and did not consider the traditional preparation methods.

Six plant species were selected for screening in our study, based on their ethnobotanical used. *Buchanania obovata* Engl. (family Anacardiaceae; commonly known as green plum and wild mango) is a small- to medium-sized tree native to the northern regions of the Northern Territory, Australia [19]. It produces small (1–1.5 cm diameter) fleshy green fruit that are high in antioxidants. As well as being considered as nutritious food, the First Australians used *B. obovata* as a bacteriocide to treat skin diseases and as a wound antiseptic, as well as for treating eye infections and diarrhoea [9, 19]. *Casuarina equisetifolia* L. (family Casuarinaceae; commonly known as whistling tree and Australian pine tree) is native to northern regions of Australia, as well as Papua New Guinea, Southeast Asia, and the Pacific Islands. Its leaves and inner cambium are used as traditional antiseptics on Groote Eylandt [9]. *Eucalyptus tetrodonta* F. Muell. (family Myrtaceae; commonly known as Darwin stringybark and messmate) was also selected due to its traditional use as an antiseptic and to treat diarrhoea [9]. It is a medium-sized tree with rough fibrous bark. Several parts of the tree, including the inner bark, leaves, and kinos have been used traditionally as antibacterial medicines [9]. *Planchonia careya* (F. Muell.) R. Knuth. (family Lecythidaceae; commonly known as cocky apple and cockatoo apple) produces edible fruit that were prized by the First Australians. The cambium layer inside the bark and the leaves were used as medicines to treat skin diseases and diarrhoea [9]. The outer bark and the roots were also used as fish poison, indicating that they may also be useful in blocking cancer cell proliferation. *Terminalia carpentariae* C. T. White (family Combretaceae; commonly known as wild peach) is a small tree native to coastal regions of northern Australia. The edible fruit of this species have high antioxidant contents. The fruit, inner bark, and leaves were traditionally used to treat skin diseases and diarrhoea [9]. Similarly*, Vigna vexillata* (L.) A. Rich. (family Fabaceae; commonly known as wild cowpea) is a climbing plant with a wide distribution. It is native to tropical regions globally, including northern regions of Australia. The root is used as food, whilst the cambium layer and the leaves are used to treat skin disorders and diarrhoea by Groote Eylandt Aborigines [9]. The ethnobotanical uses of these plants indicate that they are good candidates for screening studies. Despite these, there are few reports confirming the bioactivities of these plants, and those studies that have been published invariably report on the activity of solvent extracts or essential oils. Studies testing these plants the way that they were originally used do not yet exist.

Diarrhoea may also be caused by nonbacterial pathogens. Gastrointestinal parasites, particularly protozoa (e.g., *Cryptosporidium* spp., *Giardia duodenalis*, *Entamoeba histolytica*, *Blastocystis* spp., and *Cyclospora cayetanensis*), also frequently cause diarrhoea. *Entamoeba histolytica* and *G. duodenalis* infections are relatively frequent, especially in socioeconomically depressed regions and in rural areas [20]. More than 280 million people per year are diagnosed with giardiasis internationally (WHO, http://www.who.int/ith/diseases/giardiasis/en/), accounting for a significant health burden. Despite this, the effects of traditional medicines on protozoal pathogens have been relatively neglected in diarrhoeal studies, with only a few studies screening Australian medicinal plants against *G. duodenalis* [12, 16]. Similarly, the effects of gastrointestinal viruses are poorly studied with very few studies reporting the potential of Australian plants to inhibit the replication of enteric viruses. The current study was undertaken to highlight plant species used by First Australian communities on Groote Eylandt against a panel of bacterial, protozoal, and viral gastrointestinal pathogens that cause diarrhoea. Furthermore, the antiproliferative activity of the extracts was also evaluated against a colorectal human cancer cell line as well as HeLa human cervical carcinoma cell line for comparison.

## 2. Materials and Methods

### 2.1. Study Site

**Figure 1:**
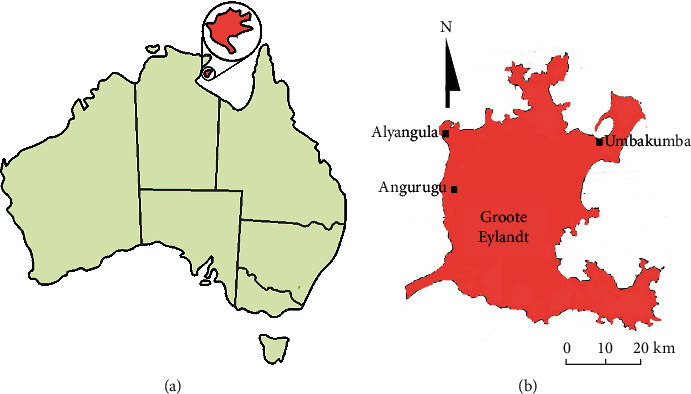
(a) The location of Groote Eylandt in relation to mainland Australia and (b) a map of Groote Eylandt showing the main settlements.

Groote Eylandt is the largest island in the Gulf of Carpentaria and lies approximately 50 km off the coast of mainland Australia (Figure 1). It is within the Arnhem Land region of Australia and experiences tropical weather conditions for the entire year. Despite its proximity to the mainland and the close relationship of the Warnindhilyagwa people with the Nunggubuyu people on the mainland, the Warnindhilyagwa tribe have retained their own unique identity and much of their traditional knowledge.

### 2.2. Plant Species Selection

Few published resources on the traditional therapeutic use of plants by the First Australians exist, and we were only able to find a single ethnobotanical book devoted to the ethnobotany of Groote Eylandt [9]. That book was used as a resource to narrow the focus and create a list of twenty plants used traditionally by the Warnindhilyagwa people to treat gastrointestinal illnesses, with an emphasis on diarrhoeal diseases. Where the usage of a plant was ambiguous, the species was excluded. This list was used to compare information obtained from informal ethnobotanical surveys conducted with a panel of Warnindhilyagwa elders. Six female tribal elders reputed to be knowledgeable about traditional medicines were surveyed to further shortlist the plants for study. The ethnobotany surveys adhered to all ethical principles in the Code of Ethics of the International Society of Ethnobotany [21]. The survey participants were asked to name plants that they knew to be useful to treat diarrhoea. Only those plant species that were named by at least four of the six survey participants were selected for further study. This listing was compared to the shortlist obtained with reference to Levitt [9], and only species on both lists were included in our study. By this process, we narrowed the focus of the study to six species.

### 2.3. Plant Material and Extraction

**Figure 2:**
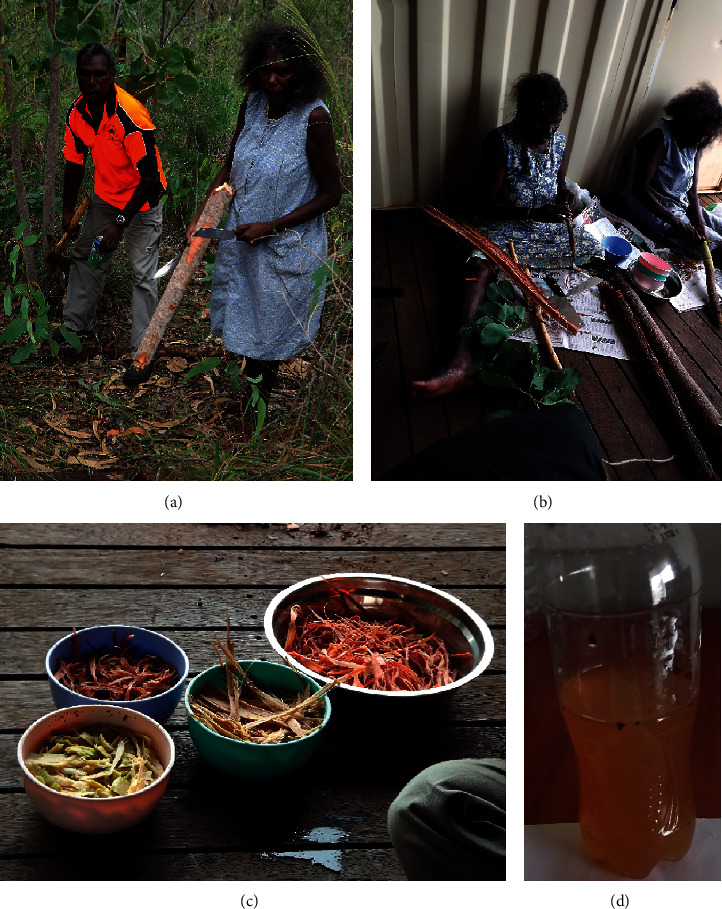
(a) Collection of plant materials on Groote Eylandt by members of the Warnindhilyagwa tribe, (b) processing of the plant material by Warnindhilyagwa elders Gayangwa Lalara and Gwen Lalara, (c) preparation of the medicines by traditional methods, and (d) an example of the traditionally prepared decoctions.

Gayangwa Lalara and Gwen Lalara, elders of the Warnindhilyagwa tribe on Groote Eylandt, were consulted to identify local plant species that were traditionally used to treat gastrointestinal diseases by the Warnindhilyagwa people. *Buchanania obovata* Engl., *Casuarina equisetifolia* L., *Eucalyptus tetrodonta* F. Muell., *Planchonia careya* (F. Muell.) R. Knuth, *Terminalia carpentariae* C. T. White, and *Vigna vexillata* (L.) A. Rich. were highlighted as useful species to screen in this study. The tribal elders identified the trees, harvested (Figure 2(a)) and processed the plant material in November 2013 (Figure 2(b)), and prepared the decoctions (Figures 2(c) and 2(d)) used in these studies. The plant species highlighted and collected on Groote Eylandt were initially identified by the tribe elders involved in the collection. High-definition photographs were taken of the entire plant as well as various morphological features (leaves, flowers, etc.) for confirmation identity. Additionally, further samples were collected for identification purposes. The identity of each species was later confirmed by botanists at the Darwin Botanical Gardens from the collected material and the photographs. Voucher specimens of all collected species are stored in the School of Environment and Science, Griffith University. All decoctions were prepared by traditional methods immediately after harvesting. The traditional preparation method was the same for all the collected materials or each plant material. Trunks of immature plant specimens were cut, and the outer bark was scraped away with a knife. The inner cambium layers were carefully collected and processed separately. The cambium of each species was pounded with the inner wood from which it was removed to soften it for extraction, and the cambium was teased apart into strands by hand to increase the surface area for extraction. Each of the plant materials was placed into individual bowls, and enough deionised water was added to just cover the plant material. The plant material was allowed to extract for an hour, and the decoction was decanted from the cambium into clean plastic bottles. The bottles were stored on ice for transport to the laboratory where they were filtered through Whatman No. 54 filter paper under vacuum. Aliquots (1 mL) of each decoction were dried in preweighed 1.5 mL microfuge tubes to determine the mass of extracted plant material/mL of decoction. The remaining aliquots of each extract were stored at −30°C until use.

### 2.4. Qualitative Phytochemical Studies

Qualitative phytochemical analysis of the plant extracts for the presence of alkaloids, anthraquinones, cardiac glycosides, flavonoids, phenolic compounds, phytosterols, saponins, tannins, and triterpenoids was evaluated by standard assays [22–24].

### 2.5. Evaluation of Antimicrobial Activity

The bioassays used to screen and quantify therapeutic properties examined in this study followed the methods of Rabadeaux et al. [25].

#### 2.5.1. Test Bacteria

*Aeromonas hydrophila* (ATCC 7966), *Bacillus cereus* (ATCC 14579), *Escherichia coli* (ATCC O157 H7), *Shigella sonnei* (ATCC 25931), *Staphylococcus aureus* (ATCC 157293), and *Streptococcus pyogenes* (ATCC 12384) were obtained from the American Type Culture Collection (ATCC), USA. The clinical bacterial strains *Alcaligenes faecalis*, *Bacillus subtilis*, *Citrobacter freundii*, *Clostridium perfringens*, *Salmonella salford*, *Salmonella newport*, *Staphylococcus epidermidis*, and *Yersinia enterocolitica* were obtained from the School of Environment and Science teaching laboratory at Griffith University. Apart from *C. perfringens*, all strains were subcultured and maintained aerobically in nutrient broth and on nutrient agar at 37°C (Oxoid Ltd., Australia). *Clostridium perfringens* was grown and maintained in thioglycolated liquid media (Oxoid Ltd., Australia) under induced anaerobic conditions in anaerobic jars using AnaeroGen™ 3.5 L atmospheric generation systems (Thermo Scientific).

#### 2.5.2. Standard Antibiotics

Penicillin G (potency of 1440–1680 *μ*g/mg), chloramphenicol (≥98% purity by HPLC), erythromycin (potency ≥850 *μ*g/mg), ciprofloxacin (≥98% purity by HPLC), and tetracycline (≥95% purity by HPLC) were purchased from Sigma-Aldrich (Australia) and were used as controls for the microplate liquid dilution assays. Standard discs of ampicillin (10 *μ*g) and chloramphenicol (10 *μ*g) were obtained from Oxoid Ltd., Australia, and served as positive controls for the disc diffusion assay.

#### 2.5.3. Evaluation of Antimicrobial Activity

The antimicrobial activity of all the plant extracts was initially determined using a modified disc diffusion method [25]. Briefly, bacterial cultures were grown in nutrient broth until they reached a count of approximately 10^8^ cells/mL; then, 100 *μ*L of each suspension was spread uniformly onto individual agar plates. The extracts were screened for antibacterial activity by applying 6 mm sterilised filter paper discs infused with 10 *μ*L of the test sample onto the agar surface and incubating for 24 hours at 37°C. Following incubation, the diameters of the inhibition zones were measured to the closest whole millimetre. Each antimicrobial assay was performed three times in triplicate (*n* = 9), and mean values were determined. Standard discs of ampicillin (10 *μ*g) and chloramphenicol (10 *μ*g) served as positive controls. Discs infused with 10 *μ*L of distilled water were included as negative controls.

#### 2.5.4. Minimum Inhibitory Concentration (MIC) Determination

The minimum inhibitory concentration for each extract was determined using two methods. Colorimetric liquid dilution MIC assays were used as they are sensitive measures of bacterial growth inhibitory activity [12]. This is a commonly used method of quantifying bacterial growth inhibition efficacy, allowing for comparisons with other studies. A solid-phase agar disc diffusion assay was also used in this study as a comparison and as a closer representation of solid-phase infections.

#### 2.5.5. Broth Microdilution MIC Assay

The MICs of the extracts were evaluated by standard broth microdilution methods [26, 27]. Briefly, overnight bacterial cultures were added dropwise to fresh nutrient broth, and the turbidity was visually adjusted to produce a McFarland number 1 standard culture. This was subsequently diluted 1 in 50 with nutrient broth, resulting in the MIC assay inoculum culture. A volume of 100 *μ*L sterile broth was added to all wells of a 96-well plate. Test extracts or control antibiotics (100 *μ*L) were then added to the top row of each plate, and doubling dilutions were prepared in each column of wells by transferring 100 *μ*L from the top well to the next well in each column, etc. A growth control (without the extract) and a sterile control (without the inoculum) were included on each plate. A volume of 100 *μ*L of respective bacterial culture was added to all wells except the sterile control wells and incubated at 37°C for 24 h. *p*-Iodonitrotetrazolium violet (INT) was obtained from Sigma-Aldrich, Australia, and dissolved in sterile deionised water to prepare a 0.2 mg/mL INT solution. A 40 *μ*L volume of this solution was added into all wells, and the plates were incubated for further 6 h at 37°C. Following incubation, the MIC was visually determined as the lowest dose at which colour development was inhibited.

#### 2.5.6. Disc Diffusion MIC Quantification

The minimum inhibitory concentration (MIC) of the extracts was also determined by disc diffusion assays following the methods of Rabadeaux et al. [25] across a range of doses, and graphs of the zone of inhibition versus the natural log of the concentration were used to calculate the MIC values of each extract.

### 2.6. Inhibitory Bioactivity against *Giardia duodenalis* Trophozoites

#### 2.6.1. Parasite Culture

The *Giardia duodenalis* S-2 (sheep strain 2) trophozoite strain used in this study was a gift from Professor Ann McDonnell, Griffith University, Australia. The trophozoites were maintained and subcultured anaerobically at 37°C in TYI-S-33 growth media supplemented with 1% bovine bile (Sigma), 10% Serum Supreme (Cambrex Bioproducts), and 200 IU/mL penicillin/200 *μ*g/mL streptomycin (Invitrogen, USA). Confluent mid-log phase cultures were passaged every 2 days by chilling the cultures on ice for a minimum of 10 min followed by vortexing to dislodge the adherent trophozoites from the walls of the culture vessel. Fresh culture media (5 mL) were seeded with approximately 1 × 10^5^ trophozoites for each passage.

#### 2.6.2. Evaluation of Anti-Giardial Activity

Anti-Giardial activity was determined as previously described [25]. Briefly, 30 *μ*L of the test extracts or the vehicle solvent or culture media (for the negative controls) was added to trophozoite suspensions (70 *μ*L containing approximately 1 × 10^5^ trophozoites) in 96-well plates. The plates were incubated anaerobically at 37°C for 12 h in a humidified anaerobic atmosphere. CellTiter 96® AQueous One Solution Cell Proliferation Assay Reagent (20 *μ*L; Promega) was added to each well, and the plates were incubated for further 3 h. The absorbances were recorded at 490 nm using a Molecular Devices, SpectraMax M3 plate reader. All tests were performed three times in triplicate (*n* = 9). The antiproliferative activity of each test was expressed as a percentage of the negative untreated control.

### 2.7. Screen for Antiviral Bioactivity

#### 2.7.1. Viral and Bacterial Stocks

The MS2 bacteriophage and F + Amp + *E. coli* used in this study were purchased from the American Type Culture Collection (ATCC). The clinical *Staphylococcus aureus* strain used in this study was obtained from Michelle Mendell, Griffith University. A volume of 30 mL of nutrient broth (Oxoid, Australia) containing 100 *μ*g/mL ampicillin (Sigma-Aldrich, Australia) was inoculated with 1 mL of F + Amp + *E. coli* culture and incubated for 2 h at 37°C to reach the log phase. A volume of 1 mL MS2 virus stock solution (containing approximately 10^8^ plaque-forming units) was subsequently added to the bacterial culture and incubated overnight at 37°C. The following day, the bacterial cells were centrifuged at 4000 rpm for 10 min, and the supernatant (containing free MS2 phage) was collected, passed through a 22 *μ*m Sarstedt filter, and stored at 4°C until use.

#### 2.7.2. Soft-Agar Overlay

Immediately prior to the MS2 phage assay, soft-agar overlay containing 0.7% (w/v) nutrient agar, 1% (w/v) glucose, 1% (w/v) CaCl_2_, and 1% (w/v) MgSO_4_ was prepared and used immediately for the MS2 plaque inhibition assay described in the following.

#### 2.7.3. MS2 Plaque Inhibition Assay

The MS2 bacteriophage plaque reduction assay was performed as previously described [28]. Briefly, 490 *μ*L of each plant extract dilution was inoculated with 10 *μ*L of MS2 virus (containing approximately 10^10^ plaque-forming units/mL) and incubated overnight at 4°C. The solution was added to 500 *μ*L *Staphylococcus aureus* and incubated at 37°C for 20 min. The mixture was subsequently added to 3 mL soft-agar overlay and poured over premade agar plates (2.8% w/v nutrient agar). Once set, the plates were incubated overnight at 37°C. The following day, the number of plaques was counted and expressed as the % untreated control. Extract dilutions were tested, and IC_50_ was determined by linear regression. Nutrient broth was used as a negative control, whilst a *C. sinensis* water extract and UV irradiation (microwave of 10 *μ*L for 4 × 30 sec) were used as positive controls.

### 2.8. Screen for Anticancer Bioactivity

#### 2.8.1. Cancer Cell Lines

In previous studies in our group, we have studied the antiproliferative activity of plant extracts against Caco2 and HeLa (American Type Culture Collection, USA) carcinoma cell lines [25]. We followed the methods described in that study to maintain and passage the cells at 37°C, 5% CO_2_ in a humidified atmosphere, until approximately 80% confluent.

#### 2.8.2. Evaluation of Cancer Cell Antiproliferative Activity

We followed the methods of Rabadeaux et al. [25] to evaluate the antiproliferative activity of the plant extracts. Briefly, 70 *μ*L aliquots of the individual carcinoma cell suspensions were aspirated into the wells of a 96-well plate. 30 *μ*L of the plant extract dilutions or controls was added to individual wells, and the plates were incubated at 37°C, 5% CO_2_. Cisplatin (50 *μ*g/mL; Sigma-Aldrich, Australia) was used as a positive control, and fresh media were included as a negative control. Following 12-hour incubation, 20 *μ*L of CellTiter 96 AQueous One Solution (Promega, Australia) was added to each well. The absorbance was recorded at 490 nm as a measure of cellular proliferation following further 3 h incubation at 37°C. The antiproliferative activity of each test was recorded as a percentage of the negative control.

#### 2.8.3. Toxicity Evaluation

Two assay methods were used to assess the toxicity of the individual samples. The *Artemia* lethality assay (ALA) was utilised for rapid preliminary toxicity screening, whilst an MTS-based cellular viability assay was used as a cellular evaluation of toxicity.

#### 2.8.4. *Artemia franciscana* Nauplii Toxicity Screening

Toxicity was tested using a modified *A. franciscana* nauplii lethality assay [29, 30]. Briefly, 400 *μ*L of seawater containing approximately 52 (mean 51.8 *n* = 125, SD 11.2) *A. franciscana* nauplii was added to wells of a 48-well plate and immediately used for bioassay. A volume of 400 *μ*L of diluted plant extracts or the reference toxin (1000 *μ*g/mL potassium dichromate) was transferred to the wells and incubated at 25 ± 1°C under artificial light (1000 lux). A negative control (400 *μ*L seawater) was included on each plate. All treatments and controls were performed three times (*n* = 9). The wells were checked at regular intervals, and the number of dead was counted. After 24 h, all nauplii were sacrificed and counted to determine the total % mortality per well. LC_50_ with 95% confidence limits for each treatment was calculated using probit analysis.

#### 2.8.5. Cell Viability Assays

The decoctions were also screened for toxicity against human primary dermal fibroblasts (HDF). The HDF cells were obtained from the American Type Culture Collection (ATCC PCS-201-012) and were cultured and maintained in Dulbecco's modified eagle medium (DMEM; Thermo Fisher Scientific, Australia), supplemented with 10% fetal calf serum (Invitrogen), 50 *μ*g/mL streptomycin (Sigma-Aldrich, Australia), and 50 IU/mL penicillin (Sigma-Aldrich, Australia) by standard methods [31, 32]. Briefly, the cells were maintained as monolayers in 7 mL flasks at 37°C, 5% CO_2_ in a humidified atmosphere, until approximately 80% confluent. Once confluency was achieved, 1 mL of trypsin (Sigma-Aldrich, Australia) was added to the culture flasks and incubated at 37°C, 5% CO_2_, for 15 min to dislodge the HDF cells. The cell suspensions were then transferred to a 10 mL centrifuge tube and sedimented by centrifugation. The supernatant was discarded, and the cells were resuspended in 9 mL of fresh media. Aliquots of the resuspended cells (70 *μ*L containing approximately 5000 cells) were added to individual wells of a 96-well plate. A volume of 30 *μ*L of the test extracts or cell media (for the negative control) was added to individual wells, and the plates were incubated at 37°C, 5% CO_2_ for 24 h in a humidified atmosphere. All extracts were screened at 200 *μ*g/mL. The cells were then washed in PBS (pH 7.2) to remove interference due to sample colour. A volume of 20 *μ*L of CellTiter 96 AQueous One Solution (Promega) was subsequently added to each well, and the plates were incubated for further 3 h. Absorbances were recorded at a test wavelength of 540 nm and a blank wavelength of 690 nm using a Molecular Devices, SpectraMax M3 plate reader. All tests were performed in at least triplicate, and triplicate controls were included on each plate. The % cellular viability of each test was calculated using the following formula:

(1) $$\begin{array}{c} \text{\% cellular viability}=\frac{\text{Abs test sample}-\left(\text{mean Abs control}-\text{mean Abs blank}\right)}{\left(\text{mean Abs control}-\text{mean Abs blank}\right)}. \end{array}$$

Cellular viability ≤50% of the untreated control indicated toxicity, whereas extracts or controls with >50% untreated control viability were deemed to be nontoxic.

#### 2.8.6. Statistical Analysis

Data are expressed as the mean ± SEM of three independent experiments, each with three internal replicates (*n* = 9). One-way ANOVA followed by Tukey's post hoc analysis were used to calculate statistical significance between control and treated groups with a *P* value <0.01 considered to be statistically significant.

## 3. Results

### 3.1. Qualitative Phytochemical Screening of the Traditional Medicines

The decoctions prepared traditionally from the individual cambiums yielded preparations with varying concentrations (Table 1). The concentrations of the decoctions were relatively consistent (generally 1–4 mg/mL) for most of the plant species. However, substantially higher concentrations were noted for the *B. obovata* and *T. carpentariae* decoctions (7.8 and 10.2 mg/mL, respectively). Phytochemical studies (Table 1) showed that all the decoctions contained similar classes of phytochemicals. All contained high relative abundances of phenolics and (with the exception of *V. vexillata*) moderate to high levels of tannins. Flavonoids were present in low to moderate abundance across all extracts, and saponins were noted in most decoctions (except *B. obovata* and *V. vexillata*). All other phytochemical classes were either missing or in low abundance in all decoctions.

### 3.2. Antibacterial Activity

**Figure 3:**
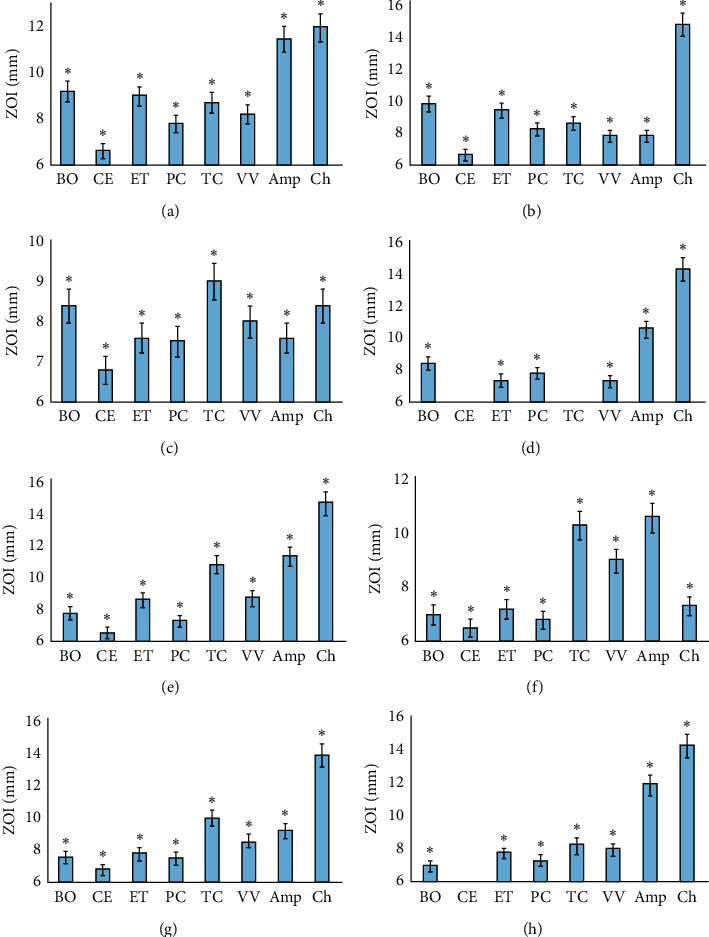
Growth inhibitory activity of the decoctions and ampicillin and chloramphenicol controls (10 *μ*g) against the Gram-negative bacteria (a) *A. faecalis*, (b) *A. hydrophila*, (c) *C. freundii*, (d) *E. coli*, (e) *S. salford*, (f) *S. newport*, (g) *S. sonnei*, and (h) *Y. enterocolitica* measured as ZOIs (mm). BO = *B. obovata* extract; CE = *C. equisetifolia* extract; ET = *E. tetrodonta* extract; PC = *P. careya* extract; TC = *T. carpentariae* extract; VV = *V. vexillata* extract; Amp = ampicillin; Ch = chloramphenicol. Results are expressed as mean ± SEM at three determinations in triplicate (*n* = 9). ^*∗*^ indicates results that are significantly different to the untreated control (*P* < 0.01).

**Figure 4:**
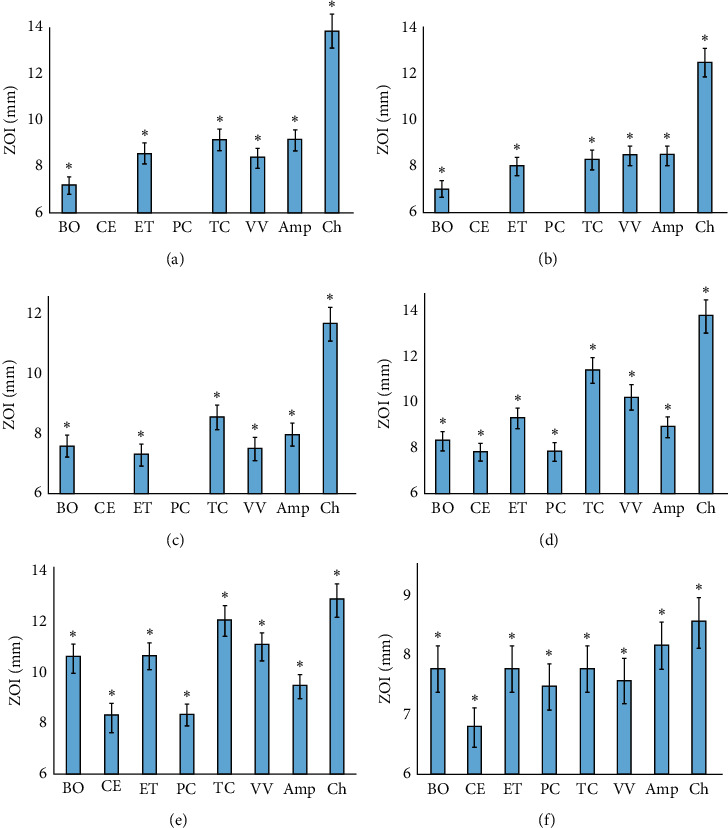
Growth inhibitory activity of the decoctions and ampicillin and chloramphenicol controls (10 *μ*g) against the Gram-positive bacteria (a) *B. cereus*, (b) *B. subtilis*, (c) *C. perfringens*, (d) *S. aureus*, (e) *S. epidermidis*, and (f) *S. pyogenes* measured as ZOIs (mm). BO = *B. obovata* extract; CE = *C. equisetifolia extract*; ET = *E. tetrodonta* extract; PC = *P. careya* extract; TC = *T. carpentariae* extract; VV = *V. vexillata* extract; Amp = ampicillin; Ch = chloramphenicol. Results are expressed as mean ± SEM at three determinations in triplicate (*n* = 9). ^*∗*^ indicates results that are significantly different to the untreated control (*P* < 0.01).

Aliquots (10 *μ*L) of each extract were tested in the disc diffusion assay against panels of Gram-negative (Figure 3) and Gram-positive bacteria (Figure 4). All the decoctions possessed broad-spectrum inhibitory activity. Indeed, the *B. obovata*, *E. tetrodonta, P. careya,* and *V. vexillata* decoctions each inhibited all the Gram-negative bacteria species tested. The *E. coli* strain was resistant to the *C. equisetifolia* and *T. carpentariae* decoctions, although all other Gram-negative bacterial species tested were inhibited by these decoctions. It is noteworthy that the *E. coli* strain tested in this study was also relatively resistant to the other decoctions as only relatively small ZOIs were measured against those decoctions. Therefore, it is possible that this is a particularly resistant strain, and other *E. coli* strains may be inhibited by these decoctions. Further work is required to test these decoctions against other *E. coli* strains.

The *B. obovata*, *E. tetrodonta, T. carpentariae*, and *V. vexillata* decoctions were also broad-spectrum inhibitors of Gram-positive bacteria, each inhibiting all of the bacteria tested. In contrast, *C. equisetifolia* and *P. careya* decoctions inhibited the growth of 50% of the Gram-positive bacterial species tested. Interestingly, both *Bacillus* spp. and *C. perfringens* were resistant to these decoctions. As both *Bacillus* and *Clostridium* genera can form endospores, it is possible that these decoctions may still be effective against the planktonic form of the bacterium yet be ineffective against the endospores. Thus, even if they kill the vegetative cells, the bacterium may regrow from spores, thus masking any inhibition. Future studies are required to specifically test these decoctions against both vegetative cells and endospores.

The relative antimicrobial potency was further evaluated by determining the MIC values (Table 2) for each extract against the bacterial species which were susceptible in the decoctions. With some notable exceptions, most of these decoctions were effective at inhibiting microbial growth at low concentrations, with liquid dilution MIC values against the bacterial species that they inhibited generally substantially <1000 *μ*g/mL, indicating the noteworthy antimicrobial activity of these preparations. The *T. carpentariae* decoction was a particularly good inhibitor of bacterial growth, with MIC values <1000 *μ*g/mL against all bacteria tested. *V. vexillata* was also a good antibacterial agent against most bacteria. Only *E. coli* had an MIC >1000 *μ*g/mL against this decoction, and its MIC of 1712 *μ*g/mL still indicates moderate activity. In contrast to the other decoctions, the *C. equisetifolia* preparation had only low to moderate antibacterial activity, with MIC values 1750–2850 *μ*g/mL. Whilst these values indicate that the *C. equisetifolia* decoction may still be useful in treating gastrointestinal bacterial infections, they would be less effective than the other extracts.

### 3.3. Anti-Giardial Activity

**Figure 5:**
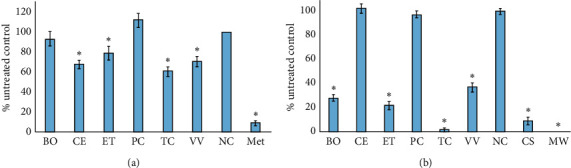
Inhibitory activity of decoctions against (a) *Giardia duodenalis* trophozoites and (b) MS2 bacteriophage measured as a percentage of the untreated control. BO = *B. obovata* decoction; CE = *C. equisetifolia* decoction; ET = *E. tetrodonta* decoction; PC = *P. careya* decoction; TC = *T. carpentariae* decoction; VV = *V. vexillata* extract; NC = negative control (seawater); Met = metronidazole, CS = *C. sinensis* control; MW = microwave control. Results are expressed as mean ± SEM of three independent experiments in triplicate (*n* = 9). ^*∗*^indicates results that are significantly different to the untreated control (*P* < 0.01).

The extracts were screened for their ability to inhibit *Giardia duodenalis* growth (Figure 5(a)). Whilst the *C. equisetifolia*, *E. tetrodonta, T. carpentariae*, and *V. vexillata* decoctions displayed significant inhibitory activity, the level of inhibition was relatively low (20–40% inhibition of control proliferation), indicating only weak growth inhibitory activity. As the inhibition of *G. duodenalis* growth did not exceed 50% at any concentration tested for these preparations, it was not possible to determine IC_50_ values for these extracts (Table 2). The *B. obovata* decoction also displayed apparent inhibition (approximately 7% inhibition) of *G. duodenalis* growth. However, this inhibition was not statistically significant.

### 3.4. Inhibition of MS2 Phage Production

Whilst MS2 phage is not infective in humans, it has previously been used as a safe and rapid test for antiviral activity against other human-infective RNA viruses [28, 33]. Thus, the MS2 phage plaque reduction assay may indicate inhibitory activity against gastrointestinal viral pathogens such as *Norovirus*. It was therefore used in this study as a preliminary screen for potential gastrointestinal antiviral activity. Four of the decoctions tested in this study displayed strong antiviral activity in this bioassay. The *T. carpentariae* decoction was a particularly good inhibitor of MS2 reproduction, inhibiting MS2 phage production by approximately 98%. This is noteworthy and is substantially more potent than the *C. sinensis* positive control (∼90% inhibition). The *T. carpentariae* decoction was also tested across a range of concentrations and an IC_50_ of 427 *μ*g/mL was determined, indicating strong antiviral activity. The *B. obovata, E. tetrodonta*, and *V. vexillata* decoctions were also good inhibitors of MS2 phage replication, inhibiting viral replication by 60–80%. The IC_50_ values determined for these decoctions (2283, 1140, and 2950 *μ*g/mL, respectively) (Table 2) also indicate noteworthy activity. In contrast, the *C. equisetifolia* and *P. careya* decoctions were completely devoid of anti-MS2 phage activity.

### 3.5. Inhibition of Cancer Cell Proliferation

**Figure 6:**
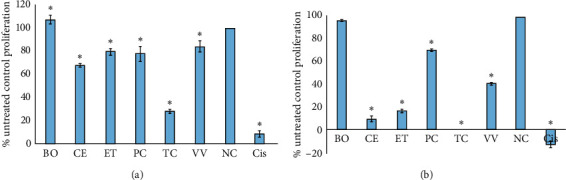
Antiproliferative activity of decoctions and controls against (a) Caco2 and (b) HeLa cancer cell lines measured as percentages of the untreated control cells. BO = *B. obovata* decoction; CE = *C. equisetifolia* decoction; ET = *E. tetrodonta*; PC = *P. careya* decoction; TC = *T. carpentariae* decoction; VV = *V. vexillata* decoction; NC = negative control (seawater); Cis = cisplatin control. Results are expressed as mean ± SEM of three independent experiments in triplicate (*n* = 9).^*∗*^indicates results that are significantly different to the untreated control (*P* < 0.01).

All decoctions were tested against 2 cancer cell lines (Caco2 human colorectal carcinoma cells, Figure 6(a); HeLa human cervical cancer cells, Figure 6(b)) to determine their effects on cell proliferation. The Caco2 cell line was selected as it is a colorectal line and thus is consistent with intestinal disease. The HeLa cells were chosen as a comparison as they are well studied and allow for comparisons with other reports. Interestingly, with the exception of *B. obovata*, all of the decoctions significantly inhibited the proliferation of both Caco2 and HeLa carcinoma cells compared to the proliferation of the untreated cells. *T. carpentariae* was a particularly strong inhibitor of Caco2 growth, inhibiting proliferation by approximately 80% compared to the untreated control cell growth (Figure 6(a)). All the other decoctions (except *B. obovata*) also significantly inhibited Caco2 proliferation, albeit by substantially lower levels (15–30%). However, it is noteworthy that the *T. carpentariae* decoction was substantially more concentrated than the other preparations, and this may account for its higher apparent activity. Inhibition of Caco2 growth by the *T. carpentariae* decoction was dose-dependent, and an IC_50_ value of 885 *μ*g/mL was determined by screening the decoction across a range of doses. We were unable to determine the IC_50_ values of any of the other preparations against Caco2 as the cell proliferation did not decrease below 50% at any concentration tested.

With the exception of the *B. obovata* preparation, the decoctions were also potent inhibitors of HeLa cell proliferation (Figure 6(b)) and were generally more potent inhibitors against this cell line than against Caco2. As for Caco2 proliferation, the *T. carpentariae* decoction was the most potent inhibitor of HeLa cell proliferation (Figure 6(b), Table 2). Indeed, the *T. carpentariae* decoction blocked 100% of the HeLa proliferation during the screening experiment, and an IC_50_ of 85 *μ*g/mL was calculated when the decoction was tested across a range of concentrations. The *C. equisetifolia*, *E. tetrodonta*, and *V. vexillata* decoctions were also strong inhibitors of HeLa cell growth, inhibiting proliferation by 60–90% (Figure 6(b)). Furthermore, IC_50_ values against HeLa of 450, 588, and 126 *μ*g/mL were calculated for these decoctions, respectively.

### 3.6. Quantification of Toxicity

**Figure 7:**
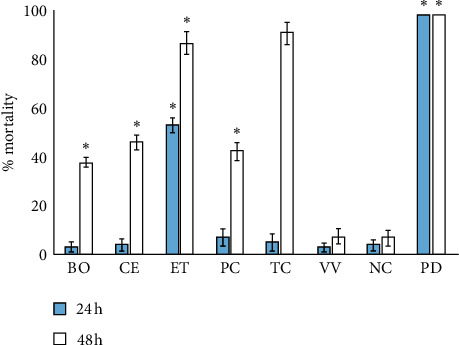
The lethality of the decoctions (1000 *μ*g/mL) and potassium dichromate control (1000 *μ*g/mL) towards *Artemia franciscana* nauplii after 24- or 48-hour exposure. BO = *B. obovata* decoction; CE = *C. equisetifolia* decoction; ET = *E. tetrodonta* decoction; PC = *P. careya* decoction; TC = *T. carpentariae* decoction; VV = *V. vexillata* decoction; NC = negative control (seawater); PD = positive control (potassium dichromate). Results are expressed as mean ± SEM of at least triplicate determinations. ^*∗*^indicates results that are significantly different to the untreated control (*P* < 0.01).

All extracts were initially screened at 1000 *μ*g/mL in the *Artemia* nauplii lethality bioassay as LC_50_ values >1000 *μ*g/mL have previously been defined as nontoxic [33]. Potassium dichromate was also included in the bioassay at 1000 *μ*g/mL as a positive control. Potassium dichromate was rapid in its induction of mortality, with significant mortality noted by 4 hours of exposure (unpublished results). The decoctions were substantially slower at inducing mortality, with ≥12 hours needed for mortality induction to become evident. Furthermore, except for the *E. tetrodonta* decoction, the traditional medicines did not induce *Artemia* nauplii mortality significantly different to that of the artificial seawater control following 24 h exposure (Figure 7). In contrast, substantial mortality was evident when nauplii were exposed to the *E. tetrodonta* decoction. As LC_50_ values >1000 *μ*g/mL have previously been defined as being nontoxic [34], only the *E. tetrodonta* decoction displayed apparent toxicity. Interestingly, the observed mortality substantially increased compared to that of the negative control for all decoctions except the *V. vexillata* decoction following 48 h exposure. As only the *E. tetrodonta* decoction induced significant mortality at 24 h, only this extract was further evaluated across a range of concentrations, and LC_50_ was determined (Table 2). Notably, LC_50_ of 1654 *μ*g/mL was determined for this decoction, and it was therefore also deemed to be nontoxic. All decoctions were also screened for toxicity in an HDF cell viability assay. Exposure of the HDF to all decoctions resulted in >50% cell viability at 200 *μ*g/mL, thus confirming that all decoctions were nontoxic.

## 4. Discussion

Recent increases in the rates of microbial resistance to clinically used antibiotics have rendered many frontline treatments to be ineffective against pathogenic diseases. This is particularly true for diarrhoea-causing pathogens. The gastrointestinal system is an ideal environment not only for microbial growth but also for the exchange of genetic information between different microbial strains, and even between different species. When a pathogen in this environment possesses antibiotic resistance genes, it can readily exchange those genes with other microbes, and individual pathogens can accumulate resistance to multiple conventional antibiotics. There is an urgent need to develop new antibiotic therapies to treat diseases caused by these pathogens. For reasons reviewed elsewhere [3], the previous methods of antibiotic discovery are unlikely to yield many new antibiotics in the future, and medical science must explore new methods to treat pathogenic diseases. A reexamination of traditional medicine is an attractive option as many traditional medicines have been used effectively for hundreds or even thousands of years. Furthermore, this use has often been well documented, allowing for selection of traditional therapies for screening. Indeed, there has been a significant increase in published studies into traditional herbal therapies to treat pathogenic diseases in most regions of the world. In contrast, the traditional remedies used by the first Australians have been relatively neglected. This may be due to a less extensive reporting of Australian Aboriginal traditional remedies, which hampers studies based on directed species selection. However, the use of plant-based medicines by some groups of first Australians, including the tribes inhabiting Groote Eylandt, has been more extensively reported, allowing for more directed studies.

This study chose plant species that were recorded as being used traditionally by the Warnindhilyagwa tribe on Groote Eylandt to treat diarrhoea and other gastrointestinal complaints. The list of identified plants was further focused following surveys and consultations with Warnindhilyagwa tribal elders. As this study aimed to validate the antipathogenic activity of the traditional Warnindhilyagwa therapies, we used decoctions prepared by the tribal elders by traditional methods and tested them undiluted, as they were used traditionally. The decoctions were initially screened against a panel of bacterial pathogens selected as they are all associated with diarrhoea and gastrointestinal disease. *Escherichia coli* is a common trigger of diarrhoea, particularly in children [35]. *Staphylococcus* spp. (including *S. aureus* and *S. epidermidis*) are common causes of antibiotic-associated nosocomial diarrhoea [36]. *Bacillus* spp. (including *B. cereus* and *B. subtilis*) and *Clostridium* spp. (including *C. perfringens*) release diarrheagenic toxins in food poisoning cases [37]. Similarly, *Shigella sonnei* can cause shigellosis [38]. A recent outbreak of fatal diarrhoea was attributed to *Aeromonas hydrophila* [39], whilst other food-borne strains including *Salmonella* spp., *A. faecalis*, and *Y. enterocolitica* [40] inhabit the lower gut and cause acute diarrhoea.

Most of the decoctions were effective at inhibiting microbial growth at low concentrations, with liquid dilution MIC values against the bacterial species that they inhibited generally substantially <1000 *μ*g/mL, indicating the noteworthy antimicrobial activity of these preparations. Interestingly, the tested decoctions were generally effective against both Gram-positive and Gram-negative bacteria, with similar efficacies. The ability of plant extracts to inhibit the growth of both Gram-positive and Gram negative-bacteria has been previously reported for other plants that have a history of medicinal usage for the treatment of microbial diseases. The antiseptic properties of the *Eucalyptus* spp. [41], *Leptospermum* spp. [42], and *Syzygium* spp. [43–47] have been studied extensively and shown to inhibit the growth of a wide variety of bacteria. However, the equal or greater susceptibility of the Gram-negative bacterial species towards the plant extracts is noteworthy. This is in contrast to other previous studies which have reported a greater susceptibility of Gram-positive bacteria towards solvent extracts for South American [6], African [48], and Australian plant extracts [43, 49], although other examples of Australian plant extracts which have a greater effects on Gram-negative bacteria have also been reported [22, 33]. The *T. carpentariae* and *V. vexillata* decoctions were particularly good antibacterial agents, with MIC values generally <1000 *μ*g/mL against most of the tested bacterial pathogens.

Whilst the MIC values reported in our study are noteworthy and validate the use of these plants for the treatment of bacterial diarrhoea, substantially higher MIC values are reported compared to several other Australian native plants. In particular, *Terminalia ferdinandiana* Excell. fruit and leaf extracts have been reported to have substantially lower MIC values against some gastrointestinal and diarrhoea-causing bacterial pathogens, with MIC values as low as 30 *μ*g/mL against some species [14, 15]. Furthermore, the *T. ferdinandiana* extracts were not only inhibitory against susceptible reference strains of *E. coli* and *S. aureus* but also had similar potency against extended spectrum beta-lactamase and methicillin-resistant strains, indicating that the extracts may function by distinct pathways, allowing them to bypass the bacterial resistance mechanisms [15]. That study identified a diversity and relative abundance of tannins, flavonoids, and terpenoids in the extracts and deduced that they contribute to their antibacterial activity. The phytochemical constituents of the decoctions tested in our study are yet to be evaluated, and future studies are planned to assess this. Furthermore, further work is required to determine whether the decoctions tested in our study are also inhibitory towards resistant bacterial pathogens.

Most previous studies screening Australian plant extracts for potential in treating gastrointestinal and diarrhoeal diseases have myopically focused on only a few common bacterial pathogens and neglected pathogens such as *C. perfringens* and *Y. enterocolitica*. However, these bacteria also cause a substantial incidence of gastrointestinal disease and should not be neglected. Notably, several of the decoctions studied herein had noteworthy activity against these bacteria. Similarly, most previous studies have also neglected testing against nonbacterial pathogens. The gastrointestinal protozoal pathogen *G. duodenalis* is particularly common and causes substantial illness globally. Indeed, approximately 280 million people per year were reported with giardiasis internationally in a 2013 study [49, 50]. All the decoctions tested in our study (except *B. obovata* and *P. careya*) significantly inhibited *G. duodenalis* proliferation when tested undiluted. However, none of the decoctions were particularly strong inhibitors of *G. duodenalis* proliferation, and we were unable to determine IC_50_ values for any extract as none inhibited proliferation by >50% at any concentration tested. This is in contrast to several other Australian plants for which good anti-Giardial activity has been reported. Notably, extracts produced from the fruit of *T. ferdinandiana* are strong inhibitors of *G. duodenalis* growth, with IC_50_ values as low as 50 *μ*g/mL [16]. It is therefore surprising that only low activity was noted for the *T. carpentariae* extract tested in this study. Notably, the previous study determined that the anti-Giardial activity of *T. ferdinandiana* corresponded to extracts rich in tannins, which are common to many *Terminalia* spp., including *T. carpentariae*. Subsequent studies have reported that the anti-Giardial activity of individual *T. ferdinandiana* components was potentiated by other extract components [18]. Thus, it is possible that even if the inhibitory components were present in the extract screened in this study, they may have only displayed weak activity if the potentiating components were absent. Of further note, fruit extracts were screened in the previous *T. ferdinandiana* studies, whereas cambium extracts were tested in this study. It is likely that the different plant parts may have substantial different phytochemical profiles, and this may account for the activity differences.

Several of the decoctions tested in this study displayed noteworthy inhibition of MS2 phage replication, with the *T. carpentariae* decoction being the most potent (IC_50_ = 427 *μ*g/mL). This may indicate that this decoction would be particularly useful in treating gastrointestinal diseases caused by viral pathogens such as *Norovirus*. These results compare favourably to other studies, which have reported antiviral activity using MS2 assays. An earlier study from our group reported comparable inhibitory activity for *Scaevola spinescens* R.Br. against the same virus and classified it as strong antiviral activity [33]. Notably, that study used *Camellia sinensis* (L.) Kuntze as the positive control and reported a substantially lower anti-MS2 phage activity (IC_50_ > 600 *μ*g/mL). Our study chose to screen the decoctions against the MS2 bacteriophage as a model for viral pathogens. Whilst this virus has previously been reported to be a good model to indicate activity against RNA viruses [28], we did not screen the decoctions against specific human viral pathogens, and further work is required to confirm the potential of these decoctions in treating viral gastrointestinal pathogens.

Antiproliferative activity against Caco2 (colorectal) and HeLa (cervical) carcinoma cell line cells was also noted for several of the decoctions, although the *T. carpentariae* decoction was the most potent inhibitor of proliferation against both cell lines, with IC_50_ values generally approximately 885 and 85 *μ*g/mL against Caco2 and HeLa cells, respectively. The antiproliferative activity of the *T. carpentariae* decoction was particularly noteworthy as colorectal cancers cause substantial mortality globally. Notably, anticancer activity has also been reported for related *Terminalia* spp. A recent study reported potent antiproliferative activity for *T. ferdinandiana* extracts, with IC_50_ values of approximately 100 *μ*g/mL against Caco2 cells, which is nearly an order of magnitude stronger than *T. carpentariae* screened in these studies [31]. Fruit and leaf extracts were tested in the *T. ferdinandiana* study, whereas cambium decoctions were tested in this study, which may account for these broad differences. Interestingly, the *T. ferdinandiana* study determined that the extracts induced their anticancer activity by elevating caspase-3 activity and inducing apoptosis. It is yet to be determined whether the same mechanisms are involved in the antiproliferative activity reported for the *T. carpentariae* decoction tested herein, and future studies are required to address this. Similarly, the *T. ferdinandiana* study also identified a diversity of tannins and flavonoids as contributing to the anticancer activity of those extracts [31]. Further study is required to determine if similar compounds contribute to the activity of the *T. carpentariae* extracts. The *C. equisetifolia* (450 *μ*g/mL)*, E. tetrodonta* (588 *μ*g/mL), and *V. vexillata* (126 *μ*g/mL) decoctions were also good inhibitors of HeLa proliferation but were substantially less effective against Caco2 cells.

Identification of the specific components responsible for the antimicrobial activity reported in the plant decoctions tested herein was beyond the scope of our study, although all decoctions were abundant in phenolics, flavonoids, and tannins. Many studies have reported potent growth inhibitory activities for a wide variety of flavonoids against extensive bacterial panels [51]. Similarly, several tannin compounds have bacterial growth inhibitory activity. Gallotannins have been reported to inhibit the growth of a broad spectrum of bacterial species [52] through a variety of mechanisms including binding cell surface molecules including lipoteichoic acid and proline-rich cell surface proteins [53, 54] and by inhibiting glucosyltransferase enzymes [55]. Ellagitannins are also highly potent inhibitors of bacterial growth, with MIC values as low as 63 *μ*g/mL [52, 54]. Ellagitannins have also been reported to function via several antibiotic mechanisms including interaction with cytoplasmic oxidoreductases and by disrupting bacterial cell walls [52, 54]. Thus, it is likely that multiple compounds within the tested decoctions contribute to the antimicrobial properties of these extracts.

The findings reported here also show that none of the decoctions displayed significant toxicity towards *A. franciscana* or HDFs. Whilst this indicates that these decoctions are safe to use therapeutically, further toxicity studies using other human cell lines are needed to further evaluate the suitability of the decoctions for medicinal purposes. The results of this study indicate that all the decoctions screened in this report are worthy of further study due to their antipathogenic activities and their abilities to block cancer cell proliferation.

## 5. Conclusions

The results of this study partially validate the traditional usage of several native Groote Eylandt plant decoctions to treat pathogenic diarrhoeal diseases and cancer, indicating that they warrant further study. The potential of *T. carpentariae* in the treatment of bacterial diarrhoea was particularly evident, with MIC values 230–350 *μ*g/mL recorded for *C. freundii*, *S. newport*, *S. sonnei*, *S. aureus*, and *S. epidermidis*. Notably, *T. carpentariae* also displayed noteworthy inhibitory activity (MICs 450–950 *μ*g/mL) against all other bacterial pathogens. *B. obovata* Engl. and *E. tetrodonta* were also good inhibitors of bacterial growth, albeit with substantially higher MIC values than determined for *T. carpentariae.* The *T. carpentariae* decoction was also the best inhibitor of MS2 phage replication (IC_50_ = 427 *μ*g/mL) and Caco2 and HeLa proliferation (IC_50_ values of 885 and 85 *μ*g/mL, respectively). None of the extracts were particularly strong inhibitors of *Giardia duodenalis* growth, indicating that none of these plants would be useful against giardiasis. From these results, *T. carpentariae* stands out as the most promising species for further study. Bioactivity-driven purifications of the active components and an examination of the mechanisms of action of these agents are required.

## Figures and Tables

**Table 1:** The concentration (mg/mL) and qualitative phytochemical screening evaluations of the plant decoctions.

Decoction concentration (mg/mL)	BO	CE	ET	PC	TC	VV
	7.8	3.4	2.9	4.2	10.2	1.2
Total phenolics	+++	++	+++	+++	+++	++
Water-soluble phenolics	+++	+++	++	+++	+++	+
Water-insoluble phenolics	+++	+++	+	+++	++	++
Cardiac glycosides	—	—	—	—	—	—
Saponins	—	++	++	+	++	—
Triterpenoids	—	—	—	—	++	—
Phytosterols	—	—	—	—	-	—
Alkaloids (Mayer test)	—	—	—	—	+	—
Alkaloids (Wagner test)	—	—	—	—	-	—
Flavonoids	++	+	+	+	++	+
Tannins	+++	++	++	+++	+++	—
Anthraquinones	—	—	—	—	—	—

+++ indicates a large response; ++ indicates a moderate response; + indicates a minor response; — indicates no response in the assay; BO = *B. obovata* extract; CE = *C. equisetifolia* extract; ET = *E. tetrodonta* extract; PC = *P. careya* extract; TC = *T. carpentariae* extract; VV = *V. vexillata* extract.

**Table 2:** The MIC values (*μ*g/mL) of the decoctions against bacteria, IC_50_ values (*μ*g/mL) against *G. duodenalis,* MS2 phage, and Caco2 and HeLa cancer cell lines, and LC_50_ values (*μ*g/mL) for *A. franciscana* nauplii and HDF bioassays.

	Bioassay		Plant species						Positive control^*∗*^
			BO	CE	ET	PC	TC	VV	
Gram-negative bacteria	*A. faecalis*	DD MIC	724	2850	854	1550	966	1280	0.31
		LD MIC	557	1938	504	977	628	870	
	*A. hydrophila*	DD MIC	645	3280	798	1274	858	1453	0.31
		LD MIC	387	2230	487	905	532	1090	
	*C. freundii*	DD MIC	972	3168	1357	1260	650	1086	0.63
		LD MIC	603	2839	923	1285	351	900	
	*E. coli*	DD MIC	1228	—	2463	1844	1275	2088	0.63
		LD MIC	1151	—	1883	1438	944	1712	
	*S. salford*	DD MIC	1056	3055	884	953	766	826	0.31
		LD MIC	676	2627	628	1077	537	576	
	*S. newport*	DD MIC	2280	2865	1650	1822	362	754	0.63
		LD MIC	2029	2235	1188	1330	260	498	
	*S. sonnei*	DD MIC	1263	2528	1050	1264	408	983	0.31
		LD MIC	973	1770	704	860	258	600	
	*Y. enterocolitica*	DD MIC	1358	—	1142	1679	985	1033	0.63
		LD MIC	1032	—	834	1847	713	846	

Gram-positive bacteria	*B. cereus*	DD MIC	1287	—	852	—	710	1055	0.31
		LD MIC	940	—	699	—	440	1013	
	*B. subtilis*	DD MIC	1532	—	1146	—	1050	942	0.63
		LD MIC	1551	—	1042	—	872	688	
	*C. perfringens*	DD MIC	1033	—	1462	—	785	1104	0.63
		LD MIC	806	—	1257	—	527	751	
	*S. aureus*	DD MIC	885	1066	750	1426	435	688	0.31
		LD MIC	788	906	510	1255	296	482	
	*S. epidermidis*	DD MIC	573	927	776	1232	326	506	0.31
		LD MIC	378	853	636	1060	231	349	
	*S. pyogenes*	DD MIC	826	1850	983	1363	785	887	0.63
		LD MIC	686	1817	826	1063	573	585	

Antiprotozoal and antiviral activity	*Giardia duodenalis* IC_50_		WND	WND	WND	WND	WND	WND	22
	MS2 phage IC50		2238	WND	1140	WND	427	2950	595

Anticancer activity	Caco2 cells IC_50_		—	WND	WND	WND	885	WND	65
	HeLa cells IC_50_		—	450	588	WND	85	126	82

Toxicity	*Artemia franciscana* nauplii 24 h LC_50_		NA	NA	1654	NA	NA	NA	126
	HDF toxicity		NT	NT	NT	NT	NT	NT	—

Numbers indicate the mean IC_50_ or LC_50_ values of three experiments performed in triplicate (*n* = 9). DD MIC = minimum inhibitory concentration determined by the disc diffusion assay; LD MIC = minimum inhibitory concentration determined by the liquid dilution assay; — indicates no significant growth inhibition/brine shrimp mortality; NA indicates that the extract was not significantly different from the negative control at any concentration tested; therefore, an IC_50_ value was not able to be determined; WND indicates IC_50_ was not determined as the amount of live *Giardia* did not reach ≤50 % at any concentration tested; NT = not toxic; BO = *B. obovata* decoction; CE = *C. equisetifolia* decoction; ET = *E. tetrodonta* decoction; PC = *P. careya* decoction; TC = *T. carpentariae* decoction; VV  = *V. vexillata* extract. ^*∗*^Positive controls used for the antibacterial, *Giardia*, MS2 phage, cancer proliferation, and toxicity assays were gentamicin, metronidazole, *C. sinensis*, cisplatin, and potassium dichromate, respectively.

## Data Availability

All data are either presented in this study or are available from the corresponding author upon request.
